# Supplementary material for: High school science fair: Ethnicity trends in student participation and experience
Source: PLoS One. 2022 Mar 23;17(3):e0264861. doi: 10.1371/journal.pone.0264861 (PMC8942272; doi:10.1371/journal.pone.0264861)
Supplement: S1 Table — (PDF) [file pone.0264861.s001.pdf]

Supplemental Table 1. Comparison of survey responses by students from 2018-19 vs. 2019-2020 vs. combined 2016-18 survey years

| Survey Questions | Answers | 2018/19 (382 students) | 2019/20 (938 students) | 2016-18 (363 students) |
|---|---|---|---|---|
| | | % Students | | |
| 1. What grade are you in? | 9th | 44.8 | 43.6 | 40.5 |
| | 10th | 31.9 | 33.8 | 32.2 |
| | 11th | 13.9 | 16.5 | 11.3 |
| | 12th | 8.6 | 5.8 | 11.0 |
| 1A. Location of high school? | Suburban | - | 70.5 | - |
| | Urban | - | 20.7 | - |
| | Rural | - | 4.8 | - |
| 2. Gender? | Female | 63.9 | 59.3 | 63.4 |
| | Male | 34.3 | 40.2 | 35.8 |
| 2A. Ethnicity most identified with? | Asian | 28.8 | 35.0 | - |
| | Black | 13.1 | 9.1 | - |
| | Hispanic | 22.8 | 17.4 | - |
| | White | 32.2 | 33.5 | - |
| | Other | 1.8 | 4.1 | - |
| 3. During high school have you carried out science fair more than once? | Yes | 29.6 | 37.5 | 31.1 |
| | No | 69.4 | 61.8 | 67.2 |
| 3A. In which science fair competitions did you compete this year? | School | 54.2 | 49.9 | - |
| | District | 9.7 | 8.6 | - |
| | Regional | 20.7 | 21.4 | - |
| | State | 8.4 | 1.5 | - |
| 4. Was your science fair project Team or Individual? | Individual | 63.1 | 58.6 | 70.2 |
| | Team | 35.9 | 37.6 | 29.2 |

| | | | | |
|---|---|---|---|---|
| 5. Was the science fair project required by your school? | Yes | 71.7 | 64.5 | 67.5 |
| | No | 14.9 | 18.2 | 16.8 |
| | Satisfied School Project | 12.0 | 14.3 | 15.2 |
| 6. Do you think science fair projects should be optional or required? (Need not be for competition.) | Optional | 74.1 | 75.4 | 74.4 |
| | Required | 25.1 | 23.1 | 25.6 |
| 8. Do you think science fair projects for competition should be optional or required? | Optional | 83.8 | 83.9 | 78.8 |
| | Required | 14.4 | 13.6 | 20.9 |
| 10. From whom do you think it would be reasonable to receive help? | 1. Parents | 77.0 | 77.4 | 78.0 |
| | 2. Siblings | 63.1 | 61.2 | 61.4 |
| | 3. Other family members | 57.6 | 54.3 | 52.9 |
| | 4. Teachers | 90.3 | 90.7 | 89.8 |
| | 5. Other students | 64.1 | 61.6 | 61.2 |
| | 6. Scientists | 70.7 | 67.7 | 70.0 |
| | 7. A paid mentor | 33.0 | 27.5 | 27.0 |
| | 8. Articles on the Internet | 78.8 | 77.1 | 78.2 |
| | Articles in books or magazines | 73.3 | 70.6 | 72.5 |
| | Other | 3.9 | 2.3 | 3.9 |
| 11. Who actually helped you? | 1. Parents | 46.3 | 48.2 | 51.0 |
| | 2. Siblings | 16.5 | 10.8 | 14.3 |
| | 3. Other family members | 4.5 | 5.4 | 7.7 |
| | 4. Teachers | 53.7 | 51.8 | 55.4 |
| | 5. Other students | 30.1 | 30.7 | 23.7 |
| | 6. Scientists | 8.6 | 8.0 | 8.0 |
| | 7. A paid mentor | 0.5 | 0.4 | 1.1 |

| | | | | |
|---|---|---|---|---|
| | 8. Articles on the Internet | 52.6 | 57.5 | 57.9 |
| | Articles in books or magazines | 23.3 | 22.7 | 24.0 |
| | Other | 5.8 | 3.4 | 5.2 |
| 12. Kind of help reasonable to expect from others? | 1. Being given the main idea | 18.3 | 18.9 | 21.2 |
| | 2. Development of the idea | 39.0 | 45.6 | 40.5 |
| | 3. Gathering background research information, or finding a research site or participants | 46.1 | 45.0 | 49.3 |
| | 4. Performing the experiments | 43.2 | 46.2 | 43.5 |
| | 5. Writing the report | 12.6 | 14.7 | 13.5 |
| | 6. Fine tuning the report after it is written | 58.9 | 62.4 | 52.3 |
| | 7. Designing the poster board and presentation | 33.5 | 35.9 | 33.1 |
| | 8. Producing charts or graphs | 23.0 | 24.4 | 19.3 |
| | 9. Coaching for the interview with judges | 63.1 | 59.1 | 58.4 |
| | 10. Copying the project from someone else | 1.6 | 2.7 | 1.4 |
| | Other | 2.4 | 2.1 | 3.3 |
| 13. What kind of help did you actually receive? | 1. Being given the main idea | 8.9 | 9.4 | 13.8 |
| | 2. Development of the idea | 25.7 | 26.8 | 26.2 |
| | 3. Gathering background research information, or finding a research site or participants | 27.2 | 25.8 | 25.9 |
| | 4. Performing the experiments | 29.3 | 28.6 | 24.5 |
| | 5. Writing the report | 6.8 | 9.5 | 8.5 |
| | 6. Fine tuning the report after it is written | 35.9 | 33.2 | 32.2 |
| | 7. Designing the poster board and presentation | 19.9 | 22.3 | 19.0 |
| | 8. Producing charts or graphs | 11.8 | 13.9 | 7.7 |
| | 9. Coaching for the interview with judges | 19.4 | 20.9 | 22.0 |
| | 10. Copying the project from someone else | 0.3 | 0.7 | 1.1 |
| | Other | 6.5 | 6.1 | 8.8 |

| | | | | |
|---|---|---|---|---|
| 14. Did you get the kind of help you wanted from teachers? | Yes | 69.9 | 74.3 | 72.2 |
| | No | 28.0 | 24.6 | 26.7 |
| 16. Did you get the amount of help you wanted from teachers? | Yes | 65.4 | 73.1 | 74.1 |
| | No | 31.9 | 25.2 | 24.5 |
| 17. Were the results of your project as expected? | Yes | 63.6 | 65.6 | 63.1 |
| | No | 34.8 | 32.8 | 35.5 |
| 18. What obstacles did you face? | 1. Coming up with the main idea | 47.9 | 45.6 | 44.6 |
| | 2. Getting motivated to do the project | 46.1 | 43.1 | 35.5 |
| | 3. Becoming disappointed with the project | 29.1 | 25.5 | 21.8 |
| | 4. Limited resources | 39.8 | 38.1 | 36.1 |
| | 5. Limited knowledge | 29.1 | 30.9 | 30.6 |
| | 6. Limited skills | 23.6 | 24.5 | 22.0 |
| | 7. Limited cooperation | 15.4 | 12.7 | 11.6 |
| | 8. Getting organized | 23.3 | 26.8 | 21.2 |
| | 9. Time pressure | 59.2 | 62.6 | 57.3 |
| | 10. Not enough money | 21.5 | 16.7 | 18.7 |
| | 11. Results not as expected | 24.6 | 22.8 | 20.7 |
| | Other | 4.2 | 3.7 | 4.4 |
| 19. How did you overcome the obstacles? | 1. Used someone else’s main idea | 1.8 | 1.3 | 2.8 |
| | 2. Picked a familiar / interesting topic | 34.0 | 30.8 | 29.8 |
| | 3. Did more background research | 49.7 | 50.4 | 48.5 |
| | 4. Stopped working on the project for a while | 17.5 | 15.8 | 16.0 |
| | 5. Made a timeline to follow | 19.9 | 22.3 | 20.7 |
| | 6. Perseverance and self-discipline | 41.6 | 48.1 | 44.4 |
| | 7. Had someone else to keep me on track | 16.0 | 16.2 | 13.8 |
| | 8. Had someone else do the math | 1.3 | 1.6 | 1.1 |

| | | | | |
|---|---|---|---|---|
| | 9. Changed the research plan | 14.7 | 18.7 | 11.6 |
| | 10. Collected more data | 23.6 | 24.6 | 22.0 |
| | 11. Had someone else collect the data | 1.0 | 2.1 | 0.6 |
| | 12. Used someone else's data | 0.3 | 0.5 | 1.4 |
| | 13. Made up the data | 5.0 | 4.1 | 4.1 |
| | 14. Changed the hypothesis to fit the data | 4.7 | 3.8 | 4.4 |
| | 15. Changed the data to fit the hypothesis | 3.1 | 1.5 | 2.2 |
| | Other | 6.0 | 4.5 | 8.0 |
| 20. Are you interested in a career in the sciences or engineering? | Yes | 53.9 | 55.2 | 57.6 |
| | No | 17.5 | 19.0 | 15.2 |
| | Not Sure | 28.0 | 25.6 | 27.3 |
| 21. Did your science fair experience increase your interest in the sciences or engineering? | Yes | 52.4 | 55.4 | 58.7 |
| | No | 46.9 | 44.1 | 40.8 |
