# Supplementary material for: High school science fair: Ethnicity trends in student participation and experience
Source: PLoS One. 2022 Mar 23;17(3):e0264861. doi: 10.1371/journal.pone.0264861 (PMC8942272; doi:10.1371/journal.pone.0264861)
Supplement: S2 Table — (PDF) [file pone.0264861.s002.pdf]

Supplemental Table 2. Factors influencing the effect of SEF participation on Asian students' interest in S&E

| Survey Questions | Answers | SEF increased my interest in S&E | | |
|---|---|---|---|---|
| | | Yes % (#) (276 students) | No % (#) (162 students) | P value |
| Interested in a career in S&E | Yes | 77.2 (213) | 41.4 (67) | <.001 |
| Level of SEF competition? | District, Region or State | 49.3 (136) | 17.9 (29) | <.001 |
| SEF required? | Yes | 42.8 (118) | 80.2 (130) | <.001 |
| Project Team or Individual? | Individual | 65.6 (181) | 42.6 (69) | <.001 |
| Participation? | Did SEF > once | 33.0 (91) | 32.1 (52) | .851 |
| Who helped with your SEF project? (more than one answer is possible) | Parents | 48.6 (134) | 43.8 (71) | .338 |
| | Teachers | 59.8 (165) | 43.2 (70) | <.001 |
| | Scientists | 14.1 (39) | 3.7 (6) | <.001 |
| | Articles on the internet | 63.4 (175) | 54.9 (89) | .080 |
| | Articles in books or magazines | 32.2 (89) | 15.4 (25) | <.001 |
| Received kind of help needed from teachers? | Yes | 80.8 (223) | 55.6 (90) | <.001 |
| Types of help received? | Gathering background info, research site and participants | 29.0 (80) | 23.5 (38) | .208 |
| | Fine tuning the report | 36.2 (100) | 23.5 (38) | .005 |
| | Coaching for the interview | 33.0 (91) | 9.3 (15) | <.001 |
| Obstacles faced? | Getting organized | 23.2 (64) | 27.2 (44) | .352 |
| | Time Pressure | 67.4 (186) | 65.4 (106) | .675 |
| Ways to overcome obstacles? | More background research | 61.6 (170) | 45.7 (74) | .001 |
| | Made a timeline | 31.9 (88) | 16.0 (26) | <.001 |
| | Perseverance | 55.4 (153) | 38.3 (62) | <.001 |
