# Supplementary material for: High school science fair: Ethnicity trends in student participation and experience
Source: PLoS One. 2022 Mar 23;17(3):e0264861. doi: 10.1371/journal.pone.0264861 (PMC8942272; doi:10.1371/journal.pone.0264861)
Supplement: S3 Table — (PDF) [file pone.0264861.s003.pdf]

Supplemental Table 3. Factors influencing the effect of SEF participation on black students' interest in S&E

Survey Questions	Answers	SEF increased my interest in S&E		
		**Yes % (#) (63 students)**	**No % (#) (72 students)**	**P value**
Interested in a career in S&E	Yes	63.5 (40)	26.4 (19)	<.001
Level of SEF competition?	District, Region or State	25.4 (16)	5.6 (4)	.001
SEF required?	Yes	71.4 (45)	88.9 (64)	.015
Project Team or Individual?	Individual	60.3 (38)	50.0 (36)	.298
Participation?	Did SEF > once	34.9 (22)	36.1 (26)	.885
Who helped with your SEF project? (more than one answer is possible)	Parents	41.3 (26)	43.1 (31)	.860
	Teachers	61.9 (39)	33.3 (24)	.001
	Scientists	3.2 (2)	0 (0)	.215
	Articles on the internet	47.6 (30)	47.2 (34)	.963
	Articles in books or magazines	14.3 (9)	18.1 (13)	.554
Received kind of help needed from teachers?	Yes	92.1 (58)	55.6 (40)	<.001
Types of help received?	Gathering background info, research site and participants	47.6 (30)	27.8 (20)	.021
	Fine tuning the report	34.9 (22)	30.6 (22)	.589
	Coaching for the interview	25.4 (16)	6.9 (5)	.004
Obstacles faced?	Getting organized	19.0 (12)	26.4 (19)	.211
	Time Pressure	54.0 (34)	73.6 (53)	.020
Ways to overcome obstacles?	More background research	46.0 (29)	37.5 (27)	.381
	Made a timeline	25.4 (16)	13.9 (10)	.125
	Perseverance	46.0 (29)	41.7 (30)	.610
