# Supplementary material for: High school science fair: Ethnicity trends in student participation and experience
Source: PLoS One. 2022 Mar 23;17(3):e0264861. doi: 10.1371/journal.pone.0264861 (PMC8942272; doi:10.1371/journal.pone.0264861)
Supplement: S4 Table — (PDF) [file pone.0264861.s004.pdf]

Supplemental Table 4. Factors influencing the effect of SEF participation on Hispanic students' interest in S&E

Survey Questions	Answers	SEF increased my interest in S&E		
		Yes % (#) (159 students)	No % (#) (90 students)	P value
Interested in a career in S&E	Yes	67.9 (108)	37.8 (34)	<.001
Level of SEF competition?	District, Region or State	64.8 (103)	27.8 (25)	<.001
SEF required?	Yes	59.1 (94)	77.8 (70)	.003
Project Team or Individual?	Individual	66.7 (106)	56.7 (51)	.133
Participation?	Did SEF > once	49.1 (78)	27.8 (25)	.001
Who helped with your SEF project? (more than one answer is possible	Parents	47.8 (76)	28.9 (26)	.005
	Teachers	62.9 (100)	38.9 (35)	<.001
	Scientists	13.8 (22)	4.4 (4)	.029
	Articles on the internet	62.9 (100)	47.8 (43)	.024
	Articles in books or magazines	28.3 (45)	16.7 (15)	.045
Received kind of help needed from teachers?	Yes	79.2 (126)	67.8 (61)	.049
Types of help received?	Gathering background info, research site and participants	25.2 (40)	34.4 (31)	.144
	Fine tuning the report	33.3 (53)	18.9 (17)	.019
	Coaching for the interview	28.9 (46)	8.9 (8)	<.001
Obstacles faced?	Getting organized	26.4 (42)	32.2 (29)	.330
	Time Pressure	62.3 (99)	54.4 (49)	.227
Ways to overcome obstacles?	More background research	62.9 (100)	36.7 (33)	<.001
	Made a timeline	24.5 (39)	5.6 (5)	<.001
	Perseverance	5.9 (81)	3.0 (27)	.001
