# Supplementary material for: High school science fair: Ethnicity trends in student participation and experience
Source: PLoS One. 2022 Mar 23;17(3):e0264861. doi: 10.1371/journal.pone.0264861 (PMC8942272; doi:10.1371/journal.pone.0264861)
Supplement: S5 Table — (PDF) [file pone.0264861.s005.pdf]

Supplemental Table 5. Factors influencing the effect of SEF participation on white students' interest in S&E

Survey Questions	Answers	SEF increased my interest in S&E		
		Yes % (#) (198 students)	No % (#) (236 students)	P value
Interested in a career in S&E	Yes	73.2 (145)	31.4 (74)	<.001
Level of SEF competition?	District, Region or State	40.4 (80)	17.8 (42)	<.001
SEF required?	Yes	63.1 (125)	80.5 (190)	<.001
Project Team or Individual?	Individual	70.2 (139)	56.8 (134)	.005
Participation?	Did SEF > once	35.9 (71)	34.3 (81)	.738
Who helped with your SEF project? (more than one answer is possible)	Parents	56.1 (111)	52.1 (123)	.411
	Teachers	60.6 (120)	46.2 (109)	.003
	Scientists	12.1 (24)	3.0 (7)	<.001
	Articles on the internet	62.1 (123)	50.0 (118)	.012
	Articles in books or magazines	25.3 (50)	19.1 (45)	.121
Received kind of help needed from teachers?	Yes	84.3 (167)	67.8 (160)	<.001
Types of help received?	Gathering background info, research site and participants	25.3 (50)	19.1 (45)	.121
	Fine tuning the report	47.5 (94)	36.0 (85)	.019
	Coaching for the interview	22.2 (44)	14.8 (35)	.047
Obstacles faced?	Getting organized	17.2 (34)	33.1 (78)	<.001
	Time Pressure	58.1 (115)	58.5 (138)	.934
Ways to overcome obstacles?	More background research	56.1 (111)	39.8 (94)	.001
	Made a timeline	26.3 (52)	16.9 (40)	.019
	Perseverance	50.0 (99)	42.8 (101)	.134
